# Expression of CMV protein pp65 in cutaneous malignant melanoma

**DOI:** 10.1371/journal.pone.0223854

**Published:** 2019-10-11

**Authors:** Margrét Agnarsdóttir, Svetlana Popova, Irina Alafuzoff

**Affiliations:** 1 Department of Immunology, Genetics and Pathology, Rudbeck Laboratory, Uppsala University, Uppsala, Sweden; 2 Department of Clinical Pathology, Akademiska University Hospital, Uppsala, Sweden; Universidade de Sao Paulo, BRAZIL

## Abstract

Human cytomegalovirus (CVM) has been detected by immunohistochemistry (IHC) in brain tumours; however, whether CMV antigen is seen in melanomas has not yet been elucidated. Applying IHC, melanoma tissue was assessed for the expression of pp65, a tegument protein of CMV. Two cohorts were available, cohort-I and II, the latter included also related metastasis. In addition to IHC, in situ hybridisation (ISH) was carried out to assess whether CMV related genetic sequences were detectable in a subset of cases. Seventy per cent of the 142 cases in cohort-I and 50% of the 37 cases in cohort-II displayed immunoreactivity (IR). In both cohorts, the IHC outcome correlated with T-stage (Cohort I: Spearman 0.22, p = 0.01, Cohort II: Fisher exact text 0.04). In 30 of cohort-II cases, when IHC staining was carried out on both the primary tumour and the corresponding metastasis, no change in IR was noted in 53%; in 20%, the IR was lower and in 27% higher in the metastasis when compared with the primary tumour. These results were significant (Fisher exact test 0.03). Applying ISH technique on four tumour cases with detectable pp65 protein, CMV related genetic sequence was not detected. Here, we demonstrate, congruent with observations published for brain tumours, that the protein pp65 is indeed observed in substantial number of melanoma cases with IHC; however, no signal was detected with ISH technique. These findings are in line with previously reported studies, demonstrating that the role of CMV in tumours is still debatable.

## Introduction

Human cytomegalovirus (CMV) is a Herpes virus with a high seroprevalence in the general population [1–4]. In immunocompetent individuals, a primary infection is followed by life-long immunity. This virus can cause severe morbidity and mortality in immunocompromised patients e.g. transplant patients due to the potential of reactivation of the virus [5, 6].

In recent years, the virus has been implicated as being of significance in tumour progression, particularly in malignant brain tumours [7–11]. Noteworthy, several studies have been unable to reproduce these observations, as no viral proteins or genomic DNA have been detected in the tumour tissue [12, 13]. Thus, the relationship between CMV and progression of gliomas is still debated. A recent review on published literature and re-analysis claims that CMV is the cause of glioblastoma multiforme [14]. The CMV virus proteins have been reported in colon- and prostate cancer [15–17], whereas the significance of this observation is still unclear. As for malignant skin tumours, there is one study that describes human CMV DNA in non-melanoma skin cancers [18].

There are several commercial antibodies available that recognise different parts of the CMV virus. The most commonly used, also in this study, recognises the protein pp65 located in the tegument and is the most abundant CMV protein. The other main structural parts of the virus are the capsid and the envelope, whereas the tegument joins these parts to each other [2, 19]. In a previous study from our group several CMV antibodies were tested [7]. Shortly, 9 different commercial CMV antibodies were tested on a foetal brain with a confirmed CMV infection, normal brain tissue and various brains tumours. Thus we had a positive control as well as negative. All the antibodies stained positively in the foetal brain including areas with typical “owl eye” inclusions. Based on our testing Novocastras antibody pp65 was considered to perform the best (limited background, non-tumour tissue not stained) and therefore this antibody was chosen in this study.

Cutaneous malignant melanoma is, by far, the most common cause of skin related deaths in Caucasians. The incidence has increased dramatically over the last few decades [20]. The most important prognostic factor for each patient with localised disease is the tumour thickness in the skin [21–23] and the sentinel node biopsy, if applicable [24, 25]. The tumour thickness also determines the primary tumour (T) stage. Until recently, a limited number of drug therapies have been available for metastasised disease, but this has changed over the last few years with the emergence of new drugs [26–28]. Nevertheless, there is still a need to study this tumour in more detail with the aim to better understand the underlying mechanisms that influence the progression and thus maybe to identify new potential drug therapies.

The objective of this study was to assess the prevalence of human CMV virus protein pp65 in malignant melanoma tumours employing immunohistochemistry (IHC). In addition, situ hybridisation (ISH) technique was applied on selected cases to verify the outcome.

## Materials and methods

### Ethical statement

In general, when histological material is submitted for pathological examination in Sweden the patient is informed that the material will be available for further research but if the patient does not agree to that this information is recorded and the material will not be used in future research. The Regional Ethical Review Board in Uppsala, Sweden, approved the use of tissue for both cohorts employed in this study (Dnrs 2005/230, 2015/173). The Ethical Review Board decided that for Cohort I written consent from each patient was necessary but this consent was collected more than 10 years ago and that form is not available to us. For cohort II which was collected more recently employing the same research method as in cohort I written consent was not necessary as decided by the Ethical Review Board because the patient is informed when the material is submitted that it might be used in future research. For both cohorts the data was analyzed anonymously.

### Patients

Two cohorts were available for this study. Cohort-I included a total of 250 malignant melanoma tumours, including both in situ and infiltrative tumours. This cohort, collected in collaboration with the Regional Cancer Registry in Uppsala/Örebro, has been described in detail by Agnarsdottir and colleagues in 2012 [29]. Out of the 250 cases, 58 cases were excluded as they were in situ tumours, and 4 cases were excluded due to lack of information regarding the T-stage. Thus, cohort-I included a total of 188 primary infiltrative malignant melanomas, 96 of those were from men, 92 from women. The median age at diagnosis was 66 years (min/max 21/99).

Cohort-II was identified by a search in the local laboratory information system. The search was carried out for patients diagnosed during the period 2004–2013, where the goal was to identify patients with tissue samples obtained from both a primary tumour and metastasis. The following SNOMED (Systematized Nomenclature of Medicine) codes were employed: M87203 (malignant melanoma unspecified), M87213 (nodular malignant melanoma), M87433 (superficial spreading melanoma), M87423 (lentigo malignant melanoma), M87443 (acral lentiginous melanoma), M87453 (desmoplastic melanoma) and M87208 (melanoma with metastasis). A total of 92 patients were identified, excluding patients with multiple primaries in the skin and patients with non-skin primary melanoma. All the hematoxylin-eosin (HE) stained slides were re-assessed by the same pathologist (MA). The T-stage was recorded according to the 7^th^ edition of the AJCC Cancer Staging Manual [21]. Out of these 92 patients, 45 cases were excluded due to disagreement regarding the diagnosis, the sample size of the primary tumour or the metastasis was too small for tissue microarray (TMA) construction or the glass or paraffin block was not available. Thus, 47 out of 92 subjects fulfilled the search criteria with tissue available for the study. The median age at diagnosis of cohort-II was 69 years (min/max 29/98) and included 30 males and 17 females, (male/female ratio 1.8). The subtypes were as follows: superficial spreading melanoma (n = 16, 34.0%), nodular malignant melanoma (n = 17, 36.2%), lentigo maligna melanoma (n = 1, 2.1%), acral lentiginous melanoma (n = 3, 6.4%) and unspecified (n = 10, 21.3%).

In addition, a TMA including 6 normal melanocytic nevi was stained with the pp65 antibody.

### Construction of tissue microarrays

Regarding cohort-I and the TMA including normal nevi, the TMA blocks were generated as previously described [29, 30].

For cohort-II, the HE stained slides were reassessed, representative areas were identified and from each corresponding paraffin donor block, two samples 1.0 mm in diameter were transferred into the recipient block using a semiautomatic tissue arrayer (Pathology Devices, Westminster, USA).

### Immunohistochemistry

The manual immunostaining was performed as previously described [7], applying 4μm thick sections and the primary antibody NCLCMVpp65 (Novocastra, Leica Biosystems, Nussloch, Germany). Briefly, antigen retrieval was performed using Tris-EDTA buffer (pH 9.0), and endogenous peroxidase activity was blocked using 3% H_2_O_2_. Primary antibody was applied in dilution 1:200 and incubated at 4°C overnight. BrightVision detection system (Immunologic, Duiven, The Netherlands) was used as secondary reagent, together with Romulin AEC chromogen kit (BioCare Medical, Concord, CA). A positive brain sample from a foetus with a confirmed CMV infection was used as a control.

### Scoring of immunostaining

**Fig 1:**
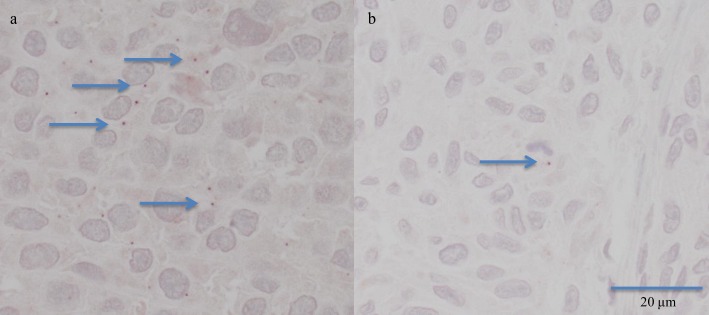
Immunohistochemistry with positive staining for pp65 in the form of small distinct granular structures, either in the nucleus or cytoplasm. In a) IR 3 (only part of the granules marked with an arrow), in b) IR 1, only 1 granule is visible (arrow), (x400).

All TMA sections were assessed systematically, and a case was included only if the core samples were representative with at least 50% of one cylinder containing tumour. Further, some cases were excluded due to heavy pigmentation of the tumour, which interfered with the assessment of IHC stain. The IHC staining outcome was assessed as previously described [7], where the intensity of the signal was evaluated. Briefly, in light microscope, in magnification x100 to x400 immunoreactivity (IR) seen either in the cytoplasm or nucleus was assessed as: 0—no IR, 1—weak IR, seen only at x400, 2—IR seen already at x200 and 3—IR seen already at x100 (Fig 1). The overall results were dichotomised to positive IR (IR 1–3) and negative IR (IR 0). One experienced pathologist (MA) annotated each cohort twice, blinded to the previous outcome; moreover, if different results were obtained, the case was evaluated for a third time and consensus was reached.

### In situ hybridisation

This analysis was performed on five tumours, four with IR 3 and one with IR 0. ZytoFast CMV probe (digoxigenin-labeled), together with ZytoFast Plus CISH Implementation Kit HRP-DAB (#T-1113-400 and #T-1063-40, ZytoVision GmBH, Bremerhaven, Germany), were used according to manufacturers instructions with minor modifications. Briefly, heat pre-treatment was performed in a decloaking chamber at temperature 98.5°C for 15 min, followed by pepsin treatment in a humidity chamber (5 min, 37°C). Specimens were air-dried, and ZytoFast CMV probe was applied. Specimens were placed into Hybridaser (Dako Cytomation, Glostrup, Denmark) for denaturation at 75°C for 10 min, and hybridisation was carried out overnight at 37°C. Thereafter, specimens were washed and positive signal was detected using DAB chromogen. A positive CMV control foetus liver (confirmed using IHC) was used. ZytoFast DNA (+) and ZytoFast DNA (-) control probes were applied as recommended by the manufacturer.

### Statistics

SPSS (IBM Analytics, New York, USA) and Microsoft excel, were used for the statistical analysis. For cohort-I, chi-square test was used for gender correlation analysis; for T-stage and age, Spearman correlation coefficient was used. Fisher exact test was used for correlation analysis of categorical variables in cohort-II. For this analysis, age was dichotomised into two groups: <70 years vs ≥ 70 years. T-stage was dichotomised into two groups: pT2a, pT2b and pT3a vs pT3b and pT4.

## Results

### Immunohistochemistry

#### Cohort-I

A total of 188 tumours were available for this study. Out of the 188 cases, 46 tumours were excluded either due to lack of tumour tissue in the stained TMA section (n = 31) or due to heavy pigmentation (n = 15) of the tumour. The staining results for the available 142 cases are given in Table 1. No IR was seen in 43 of the 142 tumours (30.3%), while in 99 cases (69.7%) the IR ranged from 1 to 3. No correlation was seen with gender (chi square 0.78, p = 0.38), or age (Spearman 0.14, p = 0.10), whereas the CMV IR (IR 0 vs IR 1–3) correlated with T-stage (Spearman 0.22, p = 0.01).

**Table 1:** CMV pp65 immunoreactivity vs T-stadium in cohort-I.

		T stadium				Total
		**1**	**2**	**3**	**4**	
**pp65**	**0**	14	11	11	7	43
**Expression**	**1**	12	20	21	19	72
	**2**	1	6	6	9	22
	**3**	0	0	3	2	5
	9*	28	12	2	4	46
**Total**		55	49	43	41	188

* 9 = results not available

#### Cohort-II

A total of 47 primary malignant melanoma tumours with a corresponding metastasis were available for this study. Out of the 47 cases, 10 tumours were excluded either due to lack of tumour tissue in the stained TMA section (n = 8) or due to heavy pigmentation (n = 2) of the tumour. The staining results for the available 37 cases are given in Table 2. No labelling (IR 0) was observed in 19 cases (51.4%), while in 18 cases (48.6%) either sparse or moderate extent of IR (1–2) was seen. No tumours displayed strong pp65 IR (IR 3). The IHC outcome was not associated with gender or age (Fisher exact text 0.17 vs 0.33, respectively), whereas the CMV IR (IR 0 vs IR 1–3) correlated with T-stage (Fisher exact text 0.04).

**Table 2:** CMV pp65 immunoreactivity vs T-stadium in cohort-II.

		T stadium				Total
		**1**	**2**	**3**	**4**	
**pp65**	**0**	0	2	12	5	19
**Expression**	**1**	0	0	2	9	11
	**2**	0	0	2	5	7
	**3**	0	0	0	0	0
	9*	0	0	6	4	10
**Total**		0	2	22	23	47

* 9 = results not available

Regarding the metastases, results were available for 39 of the 47 cases. In a set of cases, the core sample was missing from the stained TMA section (n = 5) or the tumour was too heavily pigmented (n = 3) interfering with the assessment of IHC outcome. Sixteen out of the 39 metastases (41.0%) were negative, while 23 metastases (59.0%) displayed various extent of IR (IR 1–3); noteworthy, four cases (10.3%) displayed strong IR (IR 3).

Staining results for both the primary tumour and the corresponding metastasis were available for 30 cases. The staining outcome was comparable in 16 cases (53.3%). In 6 cases (20.0%), the IR was lower and in 8 cases (26.7%) the IR was higher in the metastasis when compared with the primary tumour. When statistical analysis was carried out on dichotomised groups (IR 0 vs IR 1–3), the difference was significant (Fisher exact test 0.03).

As for the TMA including 6 normal melanocytic nevi only 3 could be annotated as the material was either lost in the block or too limited on the slide to annotate. Among the 3 cases, 2 had a weak signal, IR 1.

### In situ hybridisation

This analysis was performed on five cases, four with IR 3 and one with IR 0. No signal was detected in any of the assessed melanoma cases. The quality controls provided by the manufacturer (the positive and the negative) performed as expected and demonstrated appropriate results.

## Discussion

To our knowledge, this is the first study assessing the expression of human CMV protein pp65 with IHC technique in malignant melanoma tumours. In cohort-I 69.7% expressed pp65, most of the tumours at low or moderate levels (IR 1–2) and a few cases showed high expression (IR 3). In cohort-II, the number of positive versus negative tumours was fairly equal (48.6% vs 51.4%, respectively). These results are certainly lower when compared with results obtained while assessing malignant glial tumours collected in the same region (86–90%) [7].

In both cohorts, the results did not correlate with age or gender. Interestingly, however, the results in both cohorts correlated with T-stage, indicating that higher IR was associated with the thickness of the tumours (Cohort I: Spearman 0.22, p = 0.01, Cohort II: Fisher exact text 0.04). These results seem to be reliable as the same outcome was observed in both the small cohort-II and in the large cohort-I. Cohort-II included fewer cases when compared with cohort-I, but the tumours in cohort-II were generally thicker with higher T-stage.

In cohort-II, a cohort with a metastasis, part of the metastases displayed various extent of IR (IR 1–3, n = 23, 59%), while 16 metastases (41%) were negative. Noteworthy, four metastases (10.3%) displayed IR 3, where the corresponding primary tumour displayed a lower level of IR. The difference observed in the intensity of IHC expression between the primary tumours and the metastases was significant (Fisher exact text 0.03). This indicates that the extent of pp65 IR might differ between the primary and the metastasis, when looking at individual patients. However, the number of patients with available results for both the primary and the metastasis is limited; therefore, no reliable conclusions can be drawn. This difference might also be due to the sampling procedure, i.e. the tissue in the core of the TMA represents only part of the tumour.

As for the TMA including normal melanocytic nevi only 3 out of 6 cases could be annotated but among the 3 cases, 2 had a weak signal IR 1. However, no reliable conclusion can be drawn as the material is too limited.

The relatively high number of “lost” cores in this study was primarily due to the limited size of the tumour material to start with or to the heavy pigmentation of tumours, which interfered with the assessment of the staining.

A positive IR can be difficult to detect as a positive signal is small and easily overlooked if the IR is weak (Fig 1). The pattern of IR seen in the melanoma cohort was in line with the pattern observed in brain tumours [7]. Noteworthy the “owl eye” inclusions are seen only in active infection and we do not suggest that active infection is observed in our melanoma cases. The material in the study was annotated twice, and an experienced pathologist assessed a fraction of tumours even for a third time; thus, the assessment results can be relied upon. The main pitfall that cannot absolutely be ruled out is that the results might be altered by sampling of the tumour (tissue microarray material).

In situ hybridisation (ISH) was performed on five selected cases, four with detectable pp65 expression (IR 3) and one lacking IR (IR 0). In the assessed cases applying ISH, no signal could be detected. This observation is in line with other studies that have not been able to detect either CMV proteins or CMV related genomic DNA in brain tumours [12, 13]. Noteworthy, other reports have observed both the CMV protein and the CMV DNA or nucleic acids in the tissue specimens [8–10]. Already in 1990, when comparing ISH with IHC methods while detecting CMV and Herpes simplex viruses, it was reported that IHC was preferable due to technical difficulties regarding the ISH technique [31]. The IHC method is used to detect a protein expression in the tissue, whereas the ISH method is used to detect DNA sequences; thus, these methods are not fully comparable. The presumption is that a DNA sequence of a virus should be observed in a cell (ISH positive) and that sequence will eventually produce the protein related to the virus (IHC positive). In this study, we applied the ISH method on a limited number of tumour cases, but four out of five tested cases displayed strong and convincing IHC positivity. In total, there were only nine tumours that displayed IR 3 in the study: five primary tumours and four metastases. Although the number of cases examined with ISH is limited, they represent the tumour group most likely to be ISH positive. Negative results applying IHC or ISH methods might certainly be related to the methodology applied or to the characteristics of the tissue as previously suggested. Here, we assessed, in line with many previous studies, surgical samples that had been fixed in formalin and embedded in paraffin and used well described techniques using commercially available reagents. The methods used here are in line with those previously used. Our results are somewhat confusing as other tumour related viruses such as Epstein-Barr and Human papilloma virus have been detected in tumour tissue, both applying IHC and ISH techniques [32, 33]. In our opinion, for a convincing statement on the causal relationship between a virus and a tumour, IHC results should be confirmed with a technique detecting signs of the virus at a genomic or RNA level. Therefore, results only based on IHC must be challenged and in light of that, we conclude that the role of CMV in melanoma is still unclear.

To our knowledge, this is the first study describing expression of the pp65 CMV protein applying IHC method in malignant melanoma. The discrepant results while applying IHC and ISH are certainly disturbing and emphasise the need for further studies assessing whether CMV infection indeed has a role in the occurrence and progression of not only malignant melanomas but also other tumours.

## Supporting information

S1 Data Dataset Cohort I. (XLSX)

S2 Data Dataset Cohort II. (XLSX)
